# Risk adjustment for inter-hospital comparison of primary cesarean section rates: need, validity and parsimony

**DOI:** 10.1186/1472-6963-6-100

**Published:** 2006-08-15

**Authors:** Maria P Fantini, Elisa Stivanello, Brunella Frammartino, Anna P Barone, Danilo Fusco, Laura Dallolio, Paolo Cacciari, Carlo A Perucci

**Affiliations:** 1 Department of Medicine and Public Health, University of Bologna, Bologna, Italy; 2 Department of Epidemiology, Local Health Authority RM E, Rome, Italy; 3 Azienda Ospedaliera S. Orsola – Malpighi, University Hospital, Bologna, Italy

## Abstract

**Background:**

Cesarean section rates is often used as an indicator of quality of care in maternity hospitals. The assumption is that lower rates reflect in developed countries more appropriate clinical practice and general better performances. Hospitals are thus often ranked on the basis of caesarean section rates.

The aim of this study is to assess whether the adjustment for clinical and sociodemographic variables of the mother and the fetus is necessary for inter-hospital comparisons of cesarean section (c-section) rates and to assess whether a risk adjustment model based on a limited number of variables could be identified and used.

**Methods:**

Discharge abstracts of labouring women without prior cesarean were linked with abstracts of newborns discharged from 29 hospitals of the Emilia-Romagna Region (Italy) from 2003 to 2004. Adjusted ORs of cesarean by hospital were estimated by using two logistic regression models: 1) a full model including the potential confounders selected by a backward procedure; 2) a parsimonious model including only actual confounders identified by the "change-in-estimate" procedure. Hospital rankings, based on ORs were examined.

**Results:**

24 risk factors for c-section were included in the full model and 7 (marital status, maternal age, infant weight, fetopelvic disproportion, eclampsia or pre-eclampsia, placenta previa/abruptio placentae, malposition/malpresentation) in the parsimonious model. Hospital ranking using the adjusted ORs from both models was different from that obtained using the crude ORs. The correlation between the rankings of the two models was 0.92. The crude ORs were smaller than ORs adjusted by both models, with the parsimonious ones producing more precise estimates.

**Conclusion:**

Risk adjustment is necessary to compare hospital c-section rates, it shows differences in rankings and highlights inappropriateness of some hospitals. By adjusting for only actual confounders valid and more precise estimates could be obtained.

## Background

The evaluation of medical quality and performance has become an integral part of health care systems. Hundreds of indicators have been developed to assess the quality and performances of health facilities and health systems and to make inter-hospital comparisons. Cesarean section (c-section) rate is one of the most frequently used quality indicators to evaluate or compare obstetric departments. The utilization of c-section for reasons other than medical necessity [1-3] and the associated costs [4] make this procedure particularly subject to observation by policy makers and public health experts [5].

Hospitals and health systems are often evaluated based on their cesarean delivery rates, with the implicit assumption that lower rates reflect more appropriate, as well as more efficient, clinical practice.

Cesarean section rate has high face validity and is easy to measure because its predictors are reported in administrative data. However, the apparent simplicity of calculating the cesarean section rate can be deceptive. In fact, there is little consistency across performance measurement systems in the specifications of how it is calculated. There are differences in how the population is defined (i.e. who is included and excluded) and how the risk adjustment methodologies are applied [5].

Cesarean delivery is indicated for many clinical situations such as placenta previa, HIV and other risk factors [4,6,7] and the failure to account for such patient-specific risk factors may lead to biased comparisons. This may be particularly problematic for making inter-hospital comparisons, given the wide variations in socio-demographic and clinical characteristics of patients at different hospitals, and the referral bias that can result from differences in the availability of clinical services for women with high-risk pregnancies [8].

Risk adjustment is one technique to identify and control for potential confounders [9]. It is increasingly used in observational studies, and has already been used to compare c-section rates between hospitals that are homogenous regarding their "*a priori*" risk of cesarean delivery. Various authors have demonstrated modest to poor agreement between hospital rankings based on unadjusted and adjusted c-section rates [5,8,10-12]. Only one author did not observe important differences in hospital rankings, suggesting that additional resources for complex data adjustment may not be warranted [6].

In most cesarean c-section studies, rates were adjusted by controlling for a large number of potential confounders, including socio-demographic, maternal and fetal clinical conditions, even if they were not *actual *confounders [13]. However, collecting many variables is onerous and is associated with problems of data completeness, accuracy, and reliability, and tends to reduce the precision of the adjusted measures [14]. Moreover, no consensus has been reached regarding which clinical, demographic, and/or hospital organizational factors should be considered actual confounders.

There are only a few studies [1,10,15] regarding risk-adjustment methods for inter-hospital comparison of c-section rates that have been conducted in Europe. We present one carried out in Emilia Romagna (Italy), to determine whether risk adjustment is necessary for inter-hospital comparison and to identify a risk-adjustment model based on a limited number of variables to increase the precision of estimates without compromising validity.

## Methods

Since 1995 in the Emilia Romagna Region of Northern Italy (RER), all hospital discharge abstracts have been electronically recorded, using a Hospital Information System (HIS). The data stored in the system includes demographics [ID number, gender, date and place of birth, place of residence], discharge ID, admission and discharge dates, up to 9 discharge diagnoses and 9 procedures (*International Classification of Diseases, 9^*th *^Revision, Clinical Modification *ICD-IX-CM), ward(s) of hospitalization, date(s) of in-hospital transfer, and the regional code of the admitting facility.

We selected all hospital discharge abstracts for women in labor and of newborns from 36 maternity units in the region from January 2003 to December 2004.

This study takes as its sample live births for whom the discharge records for the mothers and infants were linked by hospital code, mother's discharge ID and date of delivery.

To identify the delivery, we used Diagnosis-Related Groups (DRGs) 370–375 from the discharge data. DRG 370 and 371 (cesarean section with and without complication, respectively) were used to identify cesarean deliveries. ICD-IX-CM diagnosis code 654.2x was used to identify any previous cesarean deliveries [16]. The number of births from primary cesareans was calculated as the difference between the number of births from c-sections deliveries and number of births from c-sections deliveries in women with previous cesareans.

Therefore, primary cesarean rates were calculated with the formula:

Number of births from primary cesarean deliveries⋅100Number of births from deliveries with no previous c-section
 MathType@MTEF@5@5@+=feaafiart1ev1aaatCvAUfKttLearuWrP9MDH5MBPbIqV92AaeXatLxBI9gBaebbnrfifHhDYfgasaacH8akY=wiFfYdH8Gipec8Eeeu0xXdbba9frFj0=OqFfea0dXdd9vqai=hGuQ8kuc9pgc9s8qqaq=dirpe0xb9q8qiLsFr0=vr0=vr0dc8meaabaqaciaacaGaaeqabaqabeGadaaakeaadaWcaaqaaiabb6eaojabbwha1jabb2gaTjabbkgaIjabbwgaLjabbkhaYjabbccaGiabb+gaVjabbAgaMjabbccaGiabbkgaIjabbMgaPjabbkhaYjabbsha0jabbIgaOjabbohaZjabbccaGiabbAgaMjabbkhaYjabb+gaVjabb2gaTjabbccaGiabbchaWjabbkhaYjabbMgaPjabb2gaTjabbggaHjabbkhaYjabbMha5jabbccaGiabbogaJjabbwgaLjabbohaZjabbggaHjabbkhaYjabbwgaLjabbggaHjabb6gaUjabbccaGiabbsgaKjabbwgaLjabbYgaSjabbMgaPjabbAha2jabbwgaLjabbkhaYjabbMgaPjabbwgaLjabbohaZjabgwSixlabigdaXiabicdaWiabicdaWaqaaiabb6eaojabbwha1jabb2gaTjabbkgaIjabbwgaLjabbkhaYjabbccaGiabb+gaVjabbAgaMjabbccaGiabbkgaIjabbMgaPjabbkhaYjabbsha0jabbIgaOjabbohaZjabbccaGiabbAgaMjabbkhaYjabb+gaVjabb2gaTjabbccaGiabbsgaKjabbwgaLjabbYgaSjabbMgaPjabbAha2jabbwgaLjabbkhaYjabbMgaPjabbwgaLjabbohaZjabbccaGiabbEha3jabbMgaPjabbsha0jabbIgaOjabbccaGiabb6gaUjabb+gaVjabbccaGiabbchaWjabbkhaYjabbwgaLjabbAha2jabbMgaPjabb+gaVjabbwha1jabbohaZjabbccaGiabbogaJjabb2caTiabbohaZjabbwgaLjabbogaJjabbsha0jabbMgaPjabb+gaVjabb6gaUbaaaaa@BCB0@

There were 62,836 births from deliveries to women with no previous c-section and they were included in our study population, excluding the following:

• mothers under 11 and over 50 years of age

• mothers discharged from hospitals without an operating room

• infants with a birth weight under 550 or over 6000 g

Hospitals with fewer than 100 deliveries per year were excluded to warrant sufficient power of comparison.

The following socio-demographic variables, considered as potential risk factors for cesarean sections, were collected: maternal age (<17, 18–20, 21–24, 25–28, 29–33, 34–38 ≥ 39), citizenship, (Italian, from developing countries, undeveloping countries other than Italy), residency (RER or other), and marital status (married, divorced-separated, single, widow). Maternal and neonatal clinical factors were also retrieved. These factors were defined using the primary and secondary discharge diagnoses of the delivery and newborn admission ' [see Additional file 1]'.

We did not consider dystocia and fetal distress as potential risk factors because of the poor reliability of their definition and because this diagnosis may reflect post ad hoc justifications of cesarean use, rather than objectively assessed conditions [17,18].

The study was conducted in collaboration with the Azienda Ospedaliera Sant'Orsola-Malpighi, the teaching hospital of the University of Bologna, Italy

### Statistical analyses

Descriptive statistics and hospital-specific crude Odds Ratios (odds of c-section for patients admitted to a specific hospital vs. odds of c-section for patients admitted to the reference category) were reported.

To take into account the role of confounders, two different logistic regression models were adopted: a "full" and a "parsimonious" model.

The "full" model was defined applying a backward selection procedure to a list of potential confounders selected according to available scientific evidence. All previously defined factors were entered and were retained if they were significant predictors of c-section (p < .05). Because of the large size of the database, an α of .05 was chosen to minimize the number of variables in the model and to maximize the strength of the association.

The "parsimonious" model was defined applying a "*change-in estimate*" procedure [19-21]. The first step of this method included the same factors entered in the full model and the exposure of interest (a specific hospital vs. reference category). Subsequently, all factors that did not modify, or only slightly modified the estimated effect of exposure, were excluded from the model.

The "*change-in estimate*" procedure identified the actual confounders for single comparisons and was repeated for each comparison (each hospital vs reference), defining as many risk adjustment models as there were comparisons. All factors, identified by at least one comparison, were included in the "parsimonious" model.

The model's performance was evaluated based on how closely it predicted the results actually observed, following the criteria for discrimination (C statistics) and calibration (Hosmer-Lemeshow test). The differences in the predictive value of the two models were assessed using the Akaike Information Criterion [22] to augment the log likelihood ratio χ^2 ^test, with a penalty for differences in the number of variables in the models compared.

The reference category included hospitals with the lowest adjusted c-section rates based on the full model. This category was defined according to the following steps:

1. 28 hospital dummies were added to the full model and the corresponding adjusted ORs were ranked. In this case the reference category was selected as the hospital with the highest number of births.

2. Four hospitals with the lowest adjusted ORs were selected as reference category.

3. Finally 25 hospital dummies, representing the rest of the 25 hospitals, were added to the full model and the estimated ORs were ranked. In this case the four hospitals, selected as reference category, were used for benchmark purposes in evaluating hospital performance for c-section in this study.

The crude and adjusted ORs obtained by the two models were used to rank hospitals, and the consistency of rankings was assessed using Spearman's rank correlation coefficient.

The statistical analysis was performed using SAS 8.2 (SAS Institute, Cary, NC) and Stata 8.2. (College Station, Texas 77845, USA).

## Results

Of a total of 62,836 births from deliveries with no previous c-sections in the RER during 2003–2004, 15,197 (24.2 %) were births from primary caesarean deliveries.

Table 1 lists the 29 hospitals involved, and their cesarean delivery rates that ranged from 11.8% to 57.0%.

Results from the multiple logistic regression models are listed in table 2. Of the twenty-four significant variables included in the full model, the greatest adjusted ORs were found for *malposition and malpresentation of fetus *(OR = 155.5; 95% CI: 126.0–190.6), *antepartum hemorrhage/abruptio placentae/placenta previa *(OR = 75.1 95% CI: 54.1–104.4), *cord prolapse *(OR = 70.3; 95% CI: 16.5–299.1) and *HIV *(OR = 28.3; 95% CI: 11.7–68.6). The following variables were not significant predictors after adjustment: pre-term delivery, premature rupture of membranes, Rh-isoimmunization and post-maturity or macrosomia.

The *change-in estimate *procedure identified seven variables which act as confounders in at least one comparison: marital status, age of mother, infant birth weight, fetopelvic disproportion/excessive development of the infant, eclampsia or pre-eclampsia, antepartum hemorrhage/abruptio placentae/placenta previa, malposition and malpresentation of fetus. These factors were included in the parsimonious model; antepartum hemorrhage/abruptio placentae/placenta previa, malposition and malpresentation of fetus accounted for the greatest adjusted ORs.

Table 3 reports the number of comparisons where each variable is identified as confounder by the *change-in estimate *procedure.

The discrimination capacities were high for both models (0.78 full model; 0.73 parsimonious model) and the Hosmer-Lemeshow statistic showed a lower calibration in the parsimonius than in the full model (H-L = 24.76 p = 0.002, H-L = 7.32 p = 0.503 respectively). The AIC statistic was similar in the two models (AIC= 52.195,9 full model; AIC= 54.803,4 parsimonious model).

Table 4 reports crude and adjusted C-section ORs for hospitals and p-values by the full and the parsimonious adjustment model.

Hospital W had the highest C-section rate when analyzing crude ORs (OR = 5.79; 95% CI: 4.83–6.94) or adjusted ORs, estimated by both models (OR = 8.77; 95% CI: 7.11–10.80 and OR = 8.55; 95% CI: 6.97–10.49 respectively). Adjusted ORs by the full model were greater than crude ORs in 24 out of 25 hospitals, adjusted ORs by the parsimonious model were greater than the crude value in 24 out of 25 hospitals

Hospital ranking using the crude and adjusted ORs is reported in table 5.

Eighteen units had their rank change after adjustment by the full model: seven facilities moved 1 position, four moved 2 or 3 positions, seven moved 4–10 positions. The parsimonious model changed the ranking of 19 facilities: ten moved 1 position, four moved 2 or 3 positions, five moved 4–10 positions.

The correlation coefficient between hospital rankings ordering crude and adjusted ORs by the full model was 0.87, and 0,80 by the parsimonious model. The correlation coefficient between hospital rankings ordering adjusted ORs by the two models was 0.92.

The ratios between upper and lower 95% confidence intervals for the hospital specific ORs estimated by the "parsimonious" model were lower than those obtained by the "full" model; the former improved the precision of the estimates.

## Discussion

Our results indicate that risk adjustment, by removing the inherent bias associated to non random allocation of deliveries, substantially changes inter-hospital comparisons.

After adjusting for heterogeneity of distribution of risk factors for c-section, the ranking of maternity units was substantially modified, with most hospitals registering higher adjusted than crude c-section rates.

In addition to adjusting for a large number of potential confounders, the specific goal of this study was to identify an efficient model that included only actual confounders of the comparison between hospitals. Including factors in a risk adjustment model that do not induce a relevant bias on the measure of association may reduce precision of estimates. The *change-in-estimate *is one method that improves the parsimony of the model and still results in precise estimates, by eliminating variables that are not actual confounders. To act as a confounder a variable must be associated with the outcome of interest (i.e. c-section) and heterogeneously distributed between categories of exposure (i.e. hospitals). Among the 24 factors identified as c-section predictors by the full model, only seven were the actual confounders used in the parsimonious model. Six out of seven actual confounders were clinical conditions of the mother (antepartum hemorrhage/abruptio placentae/placenta previa, malposition and malpresentation of fetus, eclampsia or pre-eclampsia), the fetus (birth weight), or both (fetopelvic disproportion/excessive development of the infant); these factors and maternal age have already been recognized by previous studies as risk factors for c-section [7,8,23-28]. Marital status, the remaining actual confounder, could be a surrogate of unmeasured risk. It is beyond the scope of our study to discuss the possible reasons of the heterogeneous distribution of these risk factors across hospitals, likely to be related to selection factors at work in the health care system.

Two general categories of factors might explain the variation in primary cesarean section rates between hospitals: case mix and hospital performance. In Emilia Romagna, there was an increase in inter-hospital variability after adjusting for clinical case mix, confirming that differences in c-section rates are mainly due to non clinical factors.

Although many Authors [5,11,12,18] advocate considering case mix when comparing c-section risk, the impact risk adjustment has on hospital comparisons and rankings differs between studies [5,8,28].

In addition to the mixed findings regarding the role of case mix in explaining inter-hospital variations, there are important methodological differences between studies. They are related to the source of data used (birth certificates, medical records and insurance claims), to criteria used to define c-section, to inclusion and exclusion criteria, to the final summary indicators (rates or ORs) produced, to the methods used for risk adjustment and to the variables controlled [17].

In this study we defined c-section based on DRGs. A previous study [5] showed high reliability among different methods used to calculate c-section rates.

The highest adjusted ORs obtained for the clinical variables identified as actual confounders in the parsimonious model address an important issue regarding appropriate adjustment factors. Although one would think that risk factors for cesarean section would be consistent across studies, there is inconsistency in the risk factors included in the adjustment models [8,11,29-32]. For example, factors like presentation other than vertical malposition or malpresentation, fetopelvic disproportion/excessive development, and placenta previa tend to indicate complicated pregnancies where c-section is often the only choice.

It is therefore possible that instead of adjusting for complicated pregnancies, they should be evaluated independently from others without such complications [33].

This study, as many other studies, evaluated c-section performance on administrative discharge data. Problems in accuracy, completeness, and quality might differ from hospital to hospital. The potential for inconsistencies in the coding of discharge records challenges the accuracy of the assessment of the outcome and of the risk factors in both the study population and in other populations [12]. Errors in coding could have occurred, which would have resulted in subsequent errors in adjustment. Omissions of ICD codes identifying risk factors, were more likely in the group without c-section leading to an excess of risk adjustment. Nevertheless, discharge databases are widely available at the state and regional levels, and are already routinely used. Administrative data have proved to be an accurate source to monitor c-section rates and a reliable data source to adjust for risk factors [33-36]. Moreover, administrative data from Emilia Romagna are considered of good quality, especially when compared with those from other regions in Italy. Methods used to develop models based on administrative information have the potential to be generalized to other populations. However, any risk adjustment model should be considered time and population specific.

Another limit of the study is the impossibility of including all possible clinical factors in the model; maternal parity, primipary, fetal distress and dystocia, for example are known risk factors for cesarean section [7,37], but were not included in the model because the information was either unavailable, incomplete, or considered unreliable. The result being that c-section ORs in some hospitals, especially teaching and referral hospitals might have been underestimated.

## Conclusion

Risk adjustment is necessary to compare hospital c-section rates, it shows differences in rankings and highlights inappropriateness of some hospitals. By adjusting for only actual confounders valid and more precise estimates could be obtained.

Anyway, additional studies, including qualitative studies, are recommended to identify which clinical and non clinical factors can explain inter-hospital variability. These factors should be explored in order to address the inappropriate use of this procedure.

## Competing interests

The author(s) declare that they have no competing interests.

## Authors' contributions

MPF: study conception and design, interpretation of data

ES: study design, data analysis

BF: study design, data analysis

APB: data analysis

DF: data analysis and interpretation

LD: data analysis

PC: study conception, acquisition and interpretation of data

CAP: study conception and design, interpretation of data

All authors read and approved the final manuscript.

## Pre-publication history

The pre-publication history for this paper can be accessed here:



## Supplementary Material

Additional File 1 ICD 9-CM codes identifying variables. this file contains the ICD 9-CM codes identifying variables from neonatal and maternal discharge records defined and used in the text. Click here for file

## Figures and Tables

**Table 1:** Total number of births and from primary cesarean deliveries by hospitalRER, 2003–2004

**Hospital**	**c-sections**		**Births**
	
	**N**.	**%**	**N**.
**A**	457	21.8	2092
**B**	281	23.8	1179
**C**	131	15.1	866
**D**	304	23.2	1308
**E**	387	25.7	1505
**F**	337	22.5	1496
**G**	116	29.4	395
**H**	544	20.2	2699
**I**	184	18.9	972
**J**	323	25.6	1261
**K**	126	11.8	1069
**L**	458	24.5	1867
**M**	449	24.7	1815
**N**	799	18.6	4292
**O**	200	34.6	578
**P**	134	26.6	504
**Q**	395	19.8	1996
**R**	303	15.3	1985
**S**	266	19.2	1388
**T**	597	25.7	2321
**U**	834	20.2	4121
**V**	1239	28.7	4316
**W**	299	57.0	525
**X**	111	43.2	257
**Y**	1256	29.9	4202
**Z**	913	22.2	4115
**AA**	1337	25.8	5180
**AB**	1883	29.0	6499
**AC**	534	26.3	2033
**Total**	**15197**	24.2	**62836**

**Table 2:** Crude primary c-section ORs by all variables and adjusted ORs by the variables included in the full and the parsimonious model

**VARIABLE**	**Total n**.	**% of total**	**Crude OR**	**p**	**Full Model**		**Parsimonious model**	
					
					**Adj. OR**	**p**	**Adj. OR**	**p**
*Variables from mothers' discharge records*								
AGE, yrs								
29–33	5.648	37.17	ref.		ref.		ref.	
<= 17	35	0.23	0.6	0.001	0.5	0.005	0.5	0.002
18–20	276	1.82	0.7	< 0.001	0.8	0.006	0.7	< 0.001
21–24	1.051	6.92	0.8	< 0.001	0.8	< 0.001	0.8	< 0.001
25–28	2.657	17.48	0.9	< 0.001	0.9	0.004	0.9	< 0.001
34–38	4.203	27.66	1.2	< 0.001	1.1	< 0.001	1.2	< 0.001
>=39	1.327	8.73	1.7	< 0.001	1.7	< 0.001	1.8	< 0.001
Marital status								
Married	6.700	44.09	ref.		ref.		ref.	
Divorced/separated	190	1.25	1.3	0.001	1.2	0.082	1.2	0.162
Not declared	1.124	7.40	1.0	0.224	1.1	0.048	1.1	0.011
Single	2.948	19.40	1.1	< 0.001	1.2	< 0.001	1.2	< 0.001
Widow	13	0.09	1.0	0.916	1.0	0.906	1.0	0.866
Citizenship								
Italian	12.908	84.94	ref.		ref.			
Non developing countries excluding Italy	2.193	14.43	0.9	0.489	0.8	0.103		
Developing countries	96	0.63	0.8	< 0.001	0.8	< 0.001		
Region of residence:								
RER	14.016	92.23	1.0		ref.			
Other region	1.181	7.77	0.8	< 0.001	0.7	< 0.001		
Diabetes	239	1.57	3.9	< 0.001	3.1	< 0.001		
Hypertension	505	3.32	3.4	< 0.001	3.8	< 0.001		
Lung disease	44	0.29	7.3	< 0.001	5.7	< 0.001		
HIV	38	0.25	19.9	< 0.001	28.3	< 0.001		
Other severe co-morbid illness of the mother	187	1.23	4.4	< 0.001	5.1	< 0.001		
Eclampsia or pre-eclampsia	870	5.72	9.0	< 0.001	9.1	< 0.001	8.5	< 0.001
Antepartum hemorrhage/abruptio placentae/placenta previa/	673	4.43	57.0	< 0.001	75.1	< 0.001	62.0	< 0.001
Polyhydramnios	85	0.56	5.6	< 0.001	4.3	< 0.001		
Oligohydramnios	946	6.22	2.2	< 0.001	2.9	< 0.001		
Premature rupture of membranes	1.563	10.28	0.7	< 0.001	-	-		
Other problems of the amnios	25	0.16	9.8	< 0.001	10.2	< 0.001		
Cord prolapse	33	0.22	52.0	< 0.001	70.3	< 0.001		
Abortion threads/assisted fecundation/supervision of high risk pregnancy	89	0.59	6.5	< 0.001	4.3	< 0.001		
Pre-term delivery	355	2.34	2.2	< 0.001	-	-		
Post-term delivery	424	2.73	0.8	0.001	1.4	< 0.001		
Malposition and malpresentation of fetus	3.093	20.35	124.9	< 0.001	155.5	< 0.001	145.9	< 0.001
Fetopelvic disproportion/excessive development of the infant	772	5.08	8.6	< 0.001	14.1	< 0.001	13.1	< 0.001
Fetal abnormality	127	0.84	5.1	< 0.001	3.9	< 0.001		
Rh-isoimmunization	138	0.91	0.7	< 0.001	-	-		
*Variables from neonatal discharge record*								
Infant birth weight (grams)								
> 2500	12.753	83.92	ref.		ref.		ref.	
550–1499	353	2.32	18.6	< 0.001	5.7	< 0.001	15.0	< 0.001
1500–2499	2.040	13.42	6.1	< 0.001	2.6	< 0.001	5.5	< 0.001
Congenital malformation	781	5.14	2.0	< 0.001	1.5	< 0.001		
Post-maturity or macrosomia	132	0.87	1.8	< 0.001	-	-		
*Variables from both neonatal and maternal discharge records*								
Intrauterine growth retardation	953	6.27	4.77	< 0.001	2.2	< 0.001		
Multiple pregnancy	1.323	8.71	17.29	< 0.001	9.8	< 0.001		

**Table 3:** Variables identified as confounders and occurrence of their confounding effect on total comparisons

**Variable**	**N**	**%**
Malposition and malpresentation of fetus	23	96%
Marital status	22	92%
Infant birth weight, grams	17	71%
Fetopelvic disproportion/excessive development of the infant	11	46%
Antepartum hemorrhage/abruptio placentae/placenta previa	7	29%
Eclampsia or pre-eclampsia	11	46%
Age, yrs	9	38%

**Table 4:** C-section ORs and 95% CI by hospital, obtained by crude analyses, the full and the parsimonious adjustment model

				**Full model**			**Parsimonious model**		
				
**Hospital**	**Crude OR**	**IC 95%**		**Adj OR**	**CI 95%**		**Adj OR**	**CI 95%**	
**Ref**.									
**K, Q, R, Z**									
C	0.78	0.64	0.95	1,04	0,83	1,32	0,99	0,79	1,25
**N**	1.00	0.91	1.10	1,13	1,00	1,27	1,17	1,04	1,32
**I**	1.02	0.86	1.21	1,16	0,93	1,44	1,16	0,93	1,43
**S**	1.04	0.90	1.20	1,46	1,22	1,73	1,39	1,17	1,65
**U**	1.11	1.01	1.22	1,48	1,31	1,66	1,37	1,22	1,54
**H**	1.10	0.99	1.23	1,62	1,41	1,86	1,61	1,40	1,84
**AA**	1.52	1.40	1.65	1,85	1,67	2,06	1,99	1,80	2,20
**A**	1.22	1.09	1.37	1,86	1,62	2,15	1,75	1,53	2,01
**E**	1.52	1.33	1.72	1,88	1,61	2,19	2,15	1,86	2,50
**D**	1.33	1.15	1.52	1,88	1,59	2,23	1,89	1,60	2,23
**AC**	1.56	1.39	1.74	1,90	1,65	2,18	2,02	1,77	2,32
**L**	1.42	1.26	1.60	1,93	1,67	2,24	1,82	1,58	2,10
**B**	1.37	1.19	1.58	1,96	1,65	2,34	1,93	1,63	2,29
**J**	1.51	1.31	1.73	1,97	1,66	2,33	2,16	1,84	2,55
**AB**	1.79	1.66	1.92	2,01	1,83	2,22	2,14	1,95	2,35
**F**	1.27	1.12	1.45	2,03	1,74	2,37	1,97	1,70	2,30
**Y**	1.87	1.72	2.03	2,12	1,90	2,37	2,11	1,90	2,35
**P**	1.59	1.29	1.94	2,25	1,73	2,91	2,21	1,72	2,85
**T**	1.52	1.36	1.69	2,27	1,99	2,59	2,06	1,81	2,34
**M**	1.44	1.28	1.62	2,33	2,02	2,68	2,23	1,94	2,56
**V**	1.76	1.62	1.92	2,39	2,15	2,66	2,38	2,15	2,63
**O**	2.32	1.94	2.77	2,40	1,89	3,03	2,33	1,85	2,93
**G**	1.82	1.46	2.27	2,95	2,28	3,82	2,73	2,12	3,52
**X**	3.33	2.59	4.28	4,77	3,51	6,48	4,23	3,13	5,71
**W**	5.79	4.83	6.94	8,77	7,11	10,80	8,55	6,97	10,49

**Table 5:** Hospital rank according to OR calculation methods: crude analyses, full and parsimonious model

**Hospital**	**RANK**		
	
	**Crude analyses**	**Full model**	**Parsimonious model**
**C**	1	1	1
**N**	2	2	3
**I**	3	3	2
**S**	4	4	5
**U**	6	5	4
**H**	5	6	6
**AA**	8	7	8
**A**	16	8	12
**E**	10	9	10
**D**	13	10	13
**AC**	12	11	16
**L**	20	12	7
**B**	14	13	11
**J**	7	14	19
**AB**	9	15	17
**F**	19	16	15
**Y**	11	17	9
**P**	18	18	14
**T**	21	19	18
**M**	15	20	20
**V**	23	21	22
**O**	17	22	21
**G**	22	23	23
**X**	24	24	24
**W**	25	25	25
