# Supplementary material for: Risk adjustment for inter-hospital comparison of primary cesarean section rates: need, validity and parsimony
Source: BMC Health Serv Res. 2006 Aug 15;6:100. doi: 10.1186/1472-6963-6-100 (PMC1590020; doi:10.1186/1472-6963-6-100)
Supplement: Additional File 1 — ICD 9-CM codes identifying variables. this file contains the ICD 9-CM codes identifying variables from neonatal and maternal discharge records defined and used in the text. [file 1472-6963-6-100-S1.pdf]

Additional files

A) ICD 9-CM codes identifying clinical variables from mothers' discharge records

Description	ICD 9-CM code
Diabetes	250 or 648.0, 648.8
Hypertension	40, 642.0y-642.3y (excluding when y equal to 2 or 4)
Lung disease	01, 48-51, 647.30, 647.31, 647.33, 668.01, 668.03
HIV	042 or V08
Other severe co-morbid illness of the mother	140-200, 282.4, 282.6, 286, 287, 340-342, 344, 358-359, 39, 410-430, 441, 442, 446, 580, 646.21, 646.23, 648.5, 648.6, 659.3, 669.11, 669.13, 745-747
Eclampsia or pre-eclampsia	642.4y-642.7y (excluding when y equal to 2 or 4)
Ante-partum haemorrhage/abruptio placentae/placenta previa	641
Polyhydramnios	657
Oligohydramnios	658.0
Premature rupture of membranes	658.1
Other problems of the amnios	658.4, 658.8, 658.9
Cord prolapse	663.0
Abortion threads/assisted fecundation/supervision of high risk pregnancy	630, 640, 644.0, 644.1 646.3, V26, V23
Pre-term delivery	644.2
Post-term delivery	645
Malposition and malpresentation of fetus	652
Fetopelvic disproportion/excessive development of the infant	653, 656.6
Fetal abnormality	655
RH-Isoimmunization	656.1

B) ICD 9-CM codes identifying variables from neonatal discharge records

Description	ICD 9-CM code
Congenital malformation	74, 75
Post-maturity and macrosomia	766

C) ICD 9-CM codes identifying variables from both neo and maternal discharge rec.

Description	ICD 9-CM code
Intrauterine growth retardation	764, 656.5
Multiple pregnancy	one of the following codes: 651, V27.2 –V27.9 V31-V37
